# PPM1D is a potential prognostic biomarker and correlates with immune cell infiltration in hepatocellular carcinoma

**DOI:** 10.18632/aging.203459

**Published:** 2021-09-01

**Authors:** Zhangtao Yu, Yinghui Song, Mengting Cai, Bo Jiang, Zhihua Zhang, Le Wang, Yu Jiang, Lianhong Zou, Xiehong Liu, Nanhui Yu, Xianhai Mao, Chuang Peng, Sulai Liu

**Affiliations:** 1 Department of Hepatobiliary Surgery, Hunan Research Center of Biliary Disease, The First Affiliated Hospital of Hunan Normal University/Hunan Provincial People’s Hospital, Changsha 410005, Hunan Province, China; 2 Clinical Medical Technology Research Center of Hunan Provincial for Biliary Disease Prevention and Treatment, Changsha 410005, Hunan Province, China; 3 Biliary Disease Research Laboratory of Hunan Provincial People’s Hospital, Key Laboratory of Hunan Normal University, Changsha 410005, Hunan Province, China; 4 Central Laboratory of Hunan Provincial People’s Hospital, The First Affiliated Hospital of Hunan Normal University, Changsha 410005, Hunan Province, China; 5 Department of Nuclear Medicine, Hunan Provincial People’s Hospital, The First Affiliated Hospital of Hunan Normal University, Changsha 410005, Hunan Province, China; 6 Hunan Provincial Institute of Emergency Medicine, The First Affiliated Hospital of Hunan Normal University, Hunan Provincial People’s Hospital, Changsha 410005, Hunan Province, China

**Keywords:** PPM1D, Wip1, prognostic biomarker, immune infiltrates, hepatocellular carcinoma

## Abstract

Background: Protein phosphatase magnesium-dependent 1 delta (PPM1D), also referred to as wild-type p53-induced phosphatase 1 (Wip1) or protein phosphatase 2C delta (PP2Cδ), is an oncogenic nuclear serine/threonine phosphatase belonging to the PP2C family. However, the knowledge regarding PPM1D mRNA expression, tumor immunity, and the prognosis in hepatocellular carcinoma (HCC) is scanty.

Methods: We analyzed *PPM1D*, including its expression in both the normal and tumor tissue using the Sangerbox database and Tumor Immune Estimation Resource (TIMER). We evaluated its correlation with prognosis in different tumor types by the Kaplan-Meier plotter and Gene Expression Profiling Interactive Analysis (GEPIA). The correlations between PPM1D and the cancer immune infiltrates were determined using TIMER. The correlations between PPM1D expression and gene marker sets of the immune infiltrates were established by both the TIMER and GEPIA. Immunohistochemistry was performed to detect the expression of Wip1 protein encoded by PPM1D in HCC, and the relationship between Wip1 expression and the prognosis of HCC were analyzed.

Results: We found out that PPM1D mRNA expression was significantly higher in several human cancers, including HCC, than in the corresponding normal human tissues. The PPM1D mRNA high expression in HCC was significantly correlated with poor prognosis. The expression was associated with progression-free survival (PFS) in multiple HCC patients’ cohorts (PFS HR = 1.5, *P* = 0.0066). This was especially in early stage (stage 1) and AJCC_T 1 of HCC. Besides, PPM1D mRNA expression indicated a positive correlation with tumor-infiltrating Monocytes, tumor-associated macrophages (TAMs), M1 Macrophage, M2 Macrophage, dendritic cells (DCs), T-helper (Th) and Treg. Wip1 was higher in HCC than paracancerous tissue. High expression of Wip1 was associated with poor prognosis of HCC.

Conclusion: Our findings suggested that PPM1D mRNA is critical in activating tumor immunity. Besides, they implied that PPM1D could be a potential prognostic biomarker for cancer progression. Moreover, it correlated with tumor immune cell infiltration in HCC.

## INTRODUCTION

Globally, hepatocellular carcinoma (HCC) ranks fifth in the incidence and ranks second in leading cause of cancer-related deaths of all malignant tumors [1]. There are many factors that can cause HCC, hepatitis B is well known as the main risk factor of HCC, and there are other risk factors such as alcoholic liver disease, hepatitis C and carcinogens for instance aflatoxin [2–5]. The sole radical treatment for HCC is surgical resection [6, 7]. However, most HCC patients are normally diagnosed at intermediate to advanced stages. As such they become ineligible for radical therapies. Besides, the rate of recurrence is high even with surgical resection [7]. At present, it is thus important to determine original prognostic markers and therapeutic targets for HCC.

*PPM1D* gene, encoding Wip1 phosphatase, is expressed in neutrophils, stem cells, macrophages, hematopoietic progenitors, B and T lymphocytes in peripheral blood and bone marrow [8]. Previous studies indicate that PPM1D is amplified and overexpressed in various tumors and it is hence considered an oncogene [9, 10]. PPM1D is a phosphatase which promote growth and its numerous downstream targets are important tumor promoting factors. In our previous study, we found PPM1D was overexpressed in renal clear cell carcinoma and intrahepatic cholangiocarcinoma. Its overexpression is related to poor prognosis [11–14]. It is also reported that PPM1D is one of underlying prognostic biomarkers and treatment targets for HCC [15]. However, its functions and mechanisms of HCC progression is unknown.

Based on the expression of specific markers, we studied the expression of PPM1D, its association with prognosis of HCC, while level of the various tumor-infiltrating immune cells (TIICs) in this research. Tumor Immune Estimation Resource (TIMER), Gene Expression Profiling Interactive Analysis (GEPIA) and the Kaplan-Meier plotter databases were applied in the analyses above. Our results revealed the vital contribution of PPM1D in HCC prognosis. Besides, they implied that expression of PPM1D might regulate tumor immunity through modulating the infiltration of the immune cells of HCC.

## MATERIALS AND METHODS

### PPM1D gene expression database analysis

PPM1D mRNA status of various tumors including HCC and the corresponding normal tissues were identified from the sangerbox database (http://sangerbox.com/Tool). The threshold was established with respect to the following values: P-value of 0.001, fold change of 1.5.

### Kaplan-meier plotter database analysis

Our researchers used Kaplan-Meier plotter to establish the correlation between the expression of genes and the survival rates of 21 different tumors from >10,000 tumor samples. We used the Kaplan-Meier plotter to analyse the correlation between PPM1D gene expression and the survival rates of the lung, ovarian, breast, gastric, pancreatic and liver tumors. We select the required conditions according to the default settings to get the required results; and this was foundation of hazard ratios (HR), with log-rank P-values and 95% confidence intervals.

### TIMER database analysis

TIMER (https://cistrome.shinyapps.io/timer/) is a systematic database, and we used it to analyse the TIICs in 32 tumor types from >10,000 tumor samples in The Cancer Genome Atlas (TCGA) database systematically (https://cistrome.shinyapps.io/timer/). Foundation on the investigation of the gene expression profiles, TIMER inferred the abundance of the TIICs. PPM1D expression in various tumor types was studied. We also determined the relationship between the status of PPM1D gene and the plenty of the infiltrating immune cells (macrophages, CD8+ T cells, CD4+ T cells, dendritic cells (DCs), B cells and neutrophils), according to the expression of the specific marker gene in various tumors inclusive of HCC. The gene markers for the TIICS included the markers for monocytes, neutrophils, natural killer (NK) cells, tumor-associated macrophages (TAMs), DCs, CD8+ T cells, T cells (general), B cells, T-helper 2 (Th2) cells, T-helper 1 (Th1) cells, M1 macrophages, M2 macrophages, exhausted T cells, T-helper 17 (Th17) cells, follicular helper T (Tfh) cells and regulatory T cells (Tregs). These gene markers were consulted in the prior researches [16–19].

### Gene correlation analysis in GEPIA

GEPIA database (http://gepia.cancer-pku.cn/index.html) confirmed the significantly correlated genes in TIMER. It aided in analyzing the RNA expression data of the GTEx and TCGA projects inclusive of 8,587 normal and 9,736 cancer tissue samples. Besides, GEPIA helped in generating the survival curves, which includes disease-free survival (DFS) and overall survival (OS) rates.

### Immunohistochemistry

The tissues were fixed by formalin, embedded by paraffin, and sliced into thin sections. Then, we immunostained the tissue sections with a polyclonal anti-Wip1 antibody (Santa Cruz Biotechnology, Santa Cruz, CA, USA), by using standard techniques to detect the expression of Wip1 protein [11]. Afterwards, all tissue sections were read by optical microscopies and evaluated for ≥5 fields at a × 400 magnification independently by more than two pathological professors who were not conscious of any outcome or clinical data. While the cell membrane and cytoplasm stained brown, the tissue sections were considered as wip1 positive. All images were captured by microscopic imaging system (Nikon E1000, Tokyo, Japan).

### Statistical analysis

Based on the Sangerbox and TIMER database, the gene expression analysis entailed determination of the ranks, fold changes and *P*-values. We used Kaplan-Meier plots to generate the survival curves. Gene expression corrections were performed in the GEPIA and TIMER databases by Spearman’s correlation analysis. The statically significant difference was considered when *p* < 0.05.

### Ethics approval and consent to participate

All procedures performed in studies involving human participants were in accordance with the ethical standards of the institutional and/or national research committee and with the 1964 Helsinki declaration and its later amendments or comparable ethical standards. This study was approved by the ethical committee of Hunan Normal University.

### Availability of data and materials

Data and materials are included in the manuscript.

## RESULTS

### The levels of PPM1D mRNA in various human tumors

**Figure 1:**
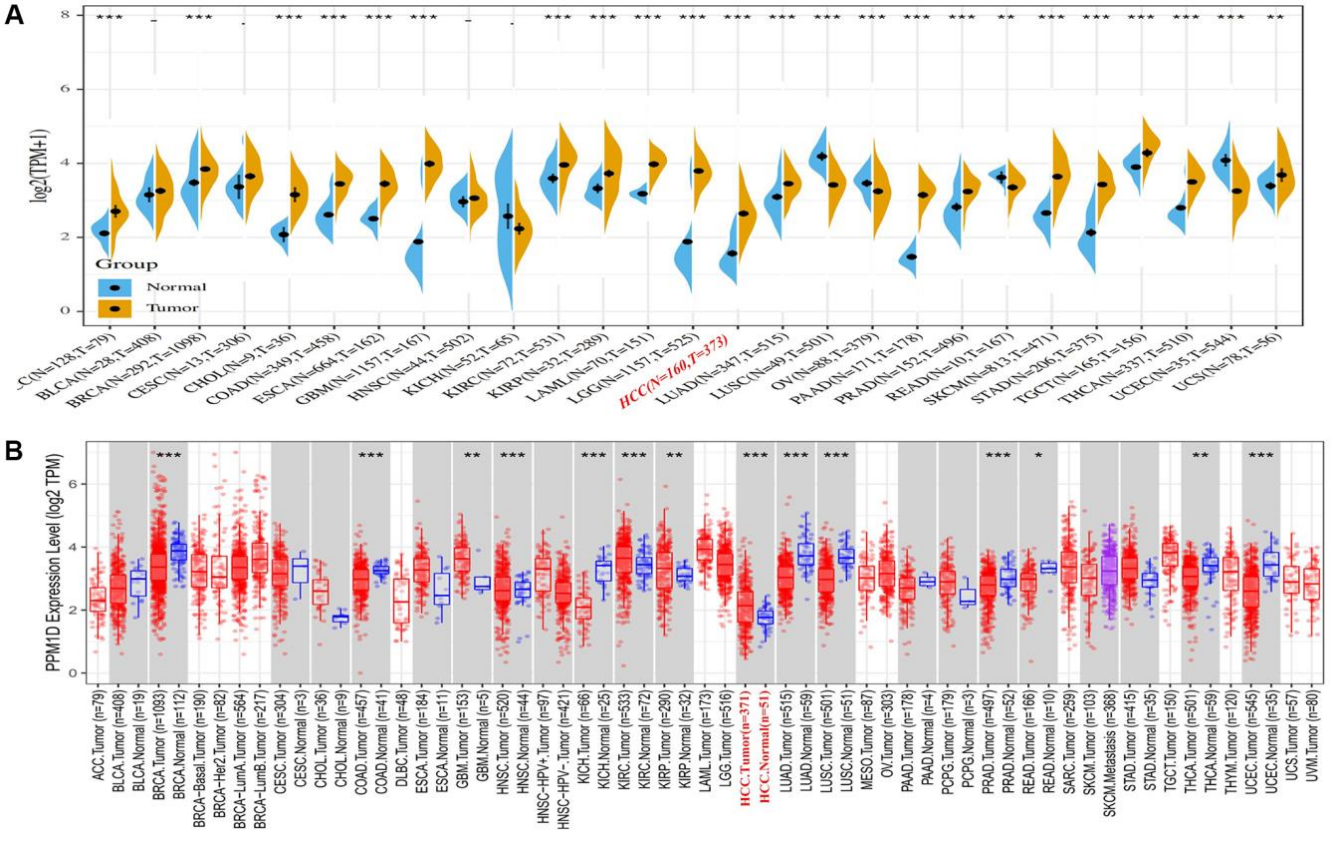
**PPM1D expression in different types of human cancers.** (**A**) High or low expression of PPM1D in different human cancer tissues compared with normal tissues using the Sangerbox database. (**B**) The level of PPM1D expression in different tumor types from the TCGA database in TIMER. Note: ^*^P < 0.05, ^**^P < 0.01, ^***^P < 0.001. Abbreviations: ACC: Adrenocortical carcinoma; BLCA: Bladder Urothelial Carcinoma; BRCA: Breast invasive carcinoma; CHOL: Cervical and endocervical cancers(CESC), Cholangiocarcinoma; COAD: Colon adenocarcinoma; DLBC: Lymphoid Neoplasm Diffuse Large B-cell Lymphoma; ESCA: Esophageal carcinoma; GBM: Glioblastoma multiforme; HNSC: Head and Neck squamous cell carcinoma; KICH: Kidney Chromophobe; KIRC: Kidney renal clear cell carcinoma; KIRP: Kidney renal papillary cell carcinoma; LAML: Acute Myeloid Leukemia; LGG: Brain Lower Grade Glioma; HCC: Hepatocellular Carcinoma; LUAD: Lung adenocarcinoma; LUSC: Lung squamous cell carcinoma; MESO: Mesothelioma; OV: Ovarian serous cystadenocarcinoma; PAAD: Pancreatic adenocarcinoma; PCPG: Pheochromocytoma and Paraganglioma; PRAD: Prostate adenocarcinoma; READ: Rectum adenocarcinoma; SKCM: Skin Cutaneous Melanoma; STAD: Stomach adenocarcinoma; TGCT: Testicular Germ Cell Tumors; THCA: Thyroid carcinoma; UCEC: Uterine Corpus Endometrial Carcinoma; UCS: Uterine Carcinosarcoma; UVM: Uveal Melanoma.

To determine the differences between PPM1D mRNA expression in normal and tumor tissues, the status of PPM1D mRNA were analyzed through Sangerbox and TIMER database. The results of the analyses demonstrated that the PPM1D expression was higher in various tumors, such as esophageal carcinoma (ESCA), glioblastoma multiforme (GBM), colon adenocarcinoma (COAD), cholangiocarcinoma (CHOL), adrenocortical carcinoma (ACC), stomach adenocarcinoma (STAD), lung adenocarcinoma (LUAD), pancreatic adenocarcinoma (PAAD), brain lower grade glioma (LGG), breast invasive carcinoma (BRCA), hepatocellular carcinoma (HCC), acute myeloid leukemia (LAML), skin cutaneous melanoma (SKCM), testicular germ cell tumors (TGCT), prostate adenocarcinoma (PRAD), kidney renal papillary cell carcinoma (KIRP), kidney renal clear cell carcinoma (KIRC), thyroid carcinoma (THCA) and uterine carcinosarcoma (UCS) tissue; and they were significantly lower in rectum adenocarcinoma (READ), uterine corpus endometrial carcinoma (UCEC), lung squamous cell carcinoma (LUSC) and ovarian serous cystadenocarcinoma (OV) than in the normal tissues, which were shown in Figure 1A. The analysis of TCGA RNA-seq data demonstrated that mRNA expression of PPM1D was significantly lower in BRCA, kidney chromophobe (KICH), lung squamous cell carcinoma (LUSC), COAD, LUAD, THCA, and UCEC comparing with the corresponding normal tissue. Though, PPM1D expression was remarkably higher in HCC in comparison to the corresponding normal tissue (Figure 1B). However, there was difference in the expression of PPM1D in some tumors, such as BRCA, COAD, LUAD and THCA, between the two databases. The results of the two databases consistently show that PPM1D was significantly extremely expressed in CHOL and HCC.

### Prognostic significance of PPM1D expression in cancers

**Figure 2:**
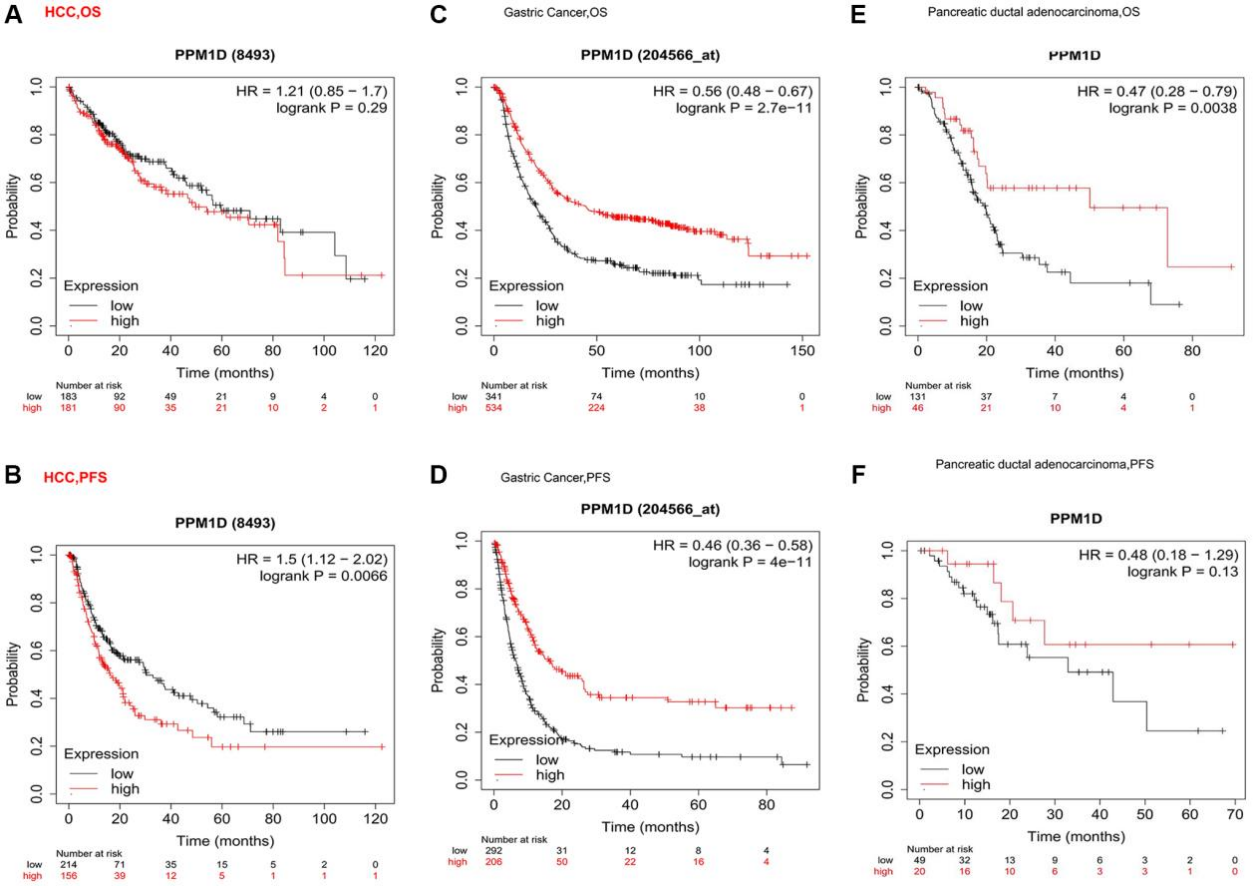
Kaplan-Meier survival curve analysis of the prognostic significance of high and low expression of PPM1D in different types of human cancers using the Kaplan-Meier plotter database (**A**–**F**). (**A**, **B**) OS and High PPM1D expression was correlated with poor PFS in HCC (*n* = 364, *n* = 370). (**C**, **D**) Survival curves of OS and PFS survival curves in the gastric cancer (*n* = 875, *n* = 498). (**E**, **F**) OS and PFS survival curves in the Pancreatic ductal adenocarcinoma (*n* = 177, *n* = 69). Abbreviations: OS: overall survival; PFS: progression-free survival.

High PPM1D expressions were not associated with OS in HCC (Figure 2A), however the mRNA expressions of PPM1D was significant related with poor prognosis in HCC (PFS: HR = 1.5, 95% CI = 1.12 to 2.02, *P* = 0.0066; Figure 2B). However, PFS in PAAD (Figure 2F) have no associated with mRNA expression of PPM1D. In gastric cancer, the high PPM1D mRNA expression was significant correlated with better prognosis (Figure 2C, 2D and 2E). These findings imply that the prognostic significance of the status of PPM1D expressions in different tumors is inconsistent (Figure 2).

In some tumors, high expression of PPM1D indicates a poor prognosis such as MESO, high expression of PPM1D in some cancers indicates a good prognosis such as KIRC, and the expression level of PPM1D in some cancers has no correlation with the prognosis such as HNSC (Supplementary Figure 1). The above results suggest that the prognostic significance of PPM1D mRNA expression in various tumors depends on tumor types.

### Association of PPM1D expressions with different clinical HCC patients through Kaplan-Meier plotter

Higher expression of PPM1D associated with poor OS, PFS, RFS and DSS (Table 1). Combined with univariate and multivariate analyses of clinicopathological factors affecting PFS of HCC patients (Table 2), we found PPM1D expression was associated with male, non-vascular infiltrating, Asian, and hepatitis virus-infected of HCC patients. In particular, the high PPM1D mRNA expression correlated with worse disease-specific (DSS), recurrence-free (RFS) and progression-free survival (PFS) in stage 1 and AJCC-T 1 of HCC patients. However, the expression of PPM1D does not seem to be related to the prognosis for the middle and late stages of HCC (Table 1).

**Table 1:** Correlation of PPM1D mRNA expression and prognosis in HCC with different characteristics by Kaplan-Meier plotter.

**Characteristics**	**OS (*n* = 364)**			**PFS (*n* = 366)**			**RFS (*n* = 313)**			**DSS (*n* = 357)**		
	***N***	**Hazard Ratio**	***P*-value**	***N***	**Hazard Ratio**	***p*-value**	***N***	**Hazard Ratio**	***p*-value**	***N***	**Hazard Ratio**	***p*-value**
**SEX**												
**Male**	246	1.87 (0.91–3.87)	0.085	246	1.52 (1.05–2.2)	**0.027**	208	1.62 (1.07–2.46)	**0.023**	241	1.87 (0.91–3.87)	0.085
**Female**	118	1.69 (0.87–3.27)	0.120	120	1.66 (0.98–2.8)	0.057	105	1.55 (0.84–2.84)	0.160	116	2.12 (1–4.45)	**0.044**
**STAGE**												
**1**	170	1.56 (0.85–2.88)	0.150	170	2.31 (1.4–3.8)	**0.000**	153	2.19 (1.27–3.76)	**0.004**	167	2.72 (1.12–6.61)	**0.021**
**2**	83	1.71 (0.68–4.28)	0.250	84	0.8 (0.42–1.51)	0.490	74	0.67 (0.34–1.29)	0.230	82	2.55 (0.56–9.52)	0.21
**3**	83	0.65 (0.36–1.18)	0.150	83	0.75 (0.44–1.29)	0.300	68	0.78 (0.42–1.47)	0.440	81	0.65 (0.32–1.34)	0.24
**4**	5	–	–	–	–	–	–	–	–	–	–	–
**AJCC_T**												
**1**	180	1.54 (0.86–2.77)	0.140	180	2.18 (1.34–3.54)	0.001	160	2.06 (1.22–3.49)	**0.006**	177	2.25 (1.01–5.04)	**0.042**
**2**	90	1.8 (0.73–4.42)	0.190	92	0.77 (0.42–1.41)	0.400	79	0.68 (0.36–1.27)	0.220	89	2.95 (0.67–12.9)	0.13
**3**	78	0.58 (0.31–1.09)	0.086	78	1.57 (0.76–3.25)	0.220	65	0.76 (0.4–1.44)	0.390	75	0.52 (0.23–1.18)	0.11
**4**	13	–	–	13	–	–	6	–	–	13	–	–
**Vascular invasion**												
**None**	203	1.72 (1.03–2.87)	0.036	204	2.11 (1.33–3.35)	**0.001**	175	1.95 (1.21–3.15)	0.006	200	1.81 (0.88–3.71)	0.099
**Micro**	90	2.14 (0.93–4.93)	0.066	91	1.41 (0.79–2.49)	0.240	81	0.52 (0.26–1.01)	0.050	88	2.45 (0.76–7.97)	0.12
**Race**												
**white**	181	0.73 (0.45–1.18)	0.190	183	1.45 (0.98–2.16)	0.060	147	1.41 (0.9–2.21)	0.130	177	1.34 (0.71–2.52)	0.37
**Asian**	155	1.9 (1.03–3.49)	**0.035**	155	1.8 (1.12–2.88)	**0.014**	143	2.01 (1.2–3.36)	**0.007**	152	5.57 (1.31–9.65)	0.0086
**Hepatitis virus**												
**Yes**	150	2.17 (1.11–4.28)	**0.021**	152	1.97 (1.24–3.12)	**0.003**	138	2.07 (1.26–3.4)	**0.003**	149	2.75 (1.13–6.69)	**0.02**
**None**	167	1.54 (0.97–2.44)	0.067	167	1.54 (0.97–2.44)	0.067	142	0.72 (0.43–1.18)	0.190	163	2 (0.99–4.05)	**0.049**

**Table 2:** Univariate and multivariate analyses of clinicopathological factors affecting PFS of HCC patients.

**Characteristics**	**Univariate analysis**		**Multivariate analysis**	
	**HR (95% CI)**	***P* value**	**HR (95% CI)**	***P* value**
SEX				
Male	1.52 (1.05–2.2)	0.027	1.42 (0.95–1.8)	0.069
Female	1.66 (0.98–2.8)	0.057	-	–
STAGE				
1	2.31(1.4–3.8)	0.00071	2.02 (1.6–3.2)	0.005
2	0.8(0.42–1.51)	0.49	–	–
3	0.75(0.44–1.29)	0.3	–	–
AJCC_T				
1	2.18 (1.34–3.54)	0.0013	1.62 (1.42–2.85)	0.02
2	0.77 (0.42–1.41)	0.4	–	–
3	1.57 (0.76–3.25)	0.22	–	–
Vascular invasion				
None	2.11 (1.33–3.35)	0.0012	1.85 (1.45–3.20)	0.034
Micro	1.41 (0.79–2.49)	0.24	–	–
Race				
White	1.45 (0.98–2.16)	0.06	–	–
Asian	1.8 (1.12–2.88)	0.014	1.62 (0.92–2.45)	0.32
Hepatitis virus				
Yes	1.97 (1.24–3.12)	0.0033	1.52 (0.67–2.86)	0.47
None	1.54 (0.97–2.44)	0.067	–	–

### The association of the levels of PPM1D with the infiltration level of immune cells in HCC

**Figure 3:**
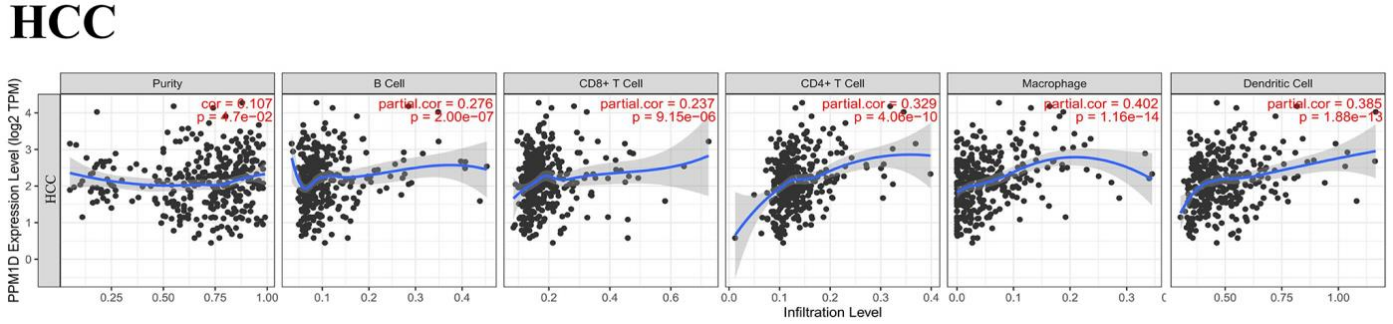
**Correlation analysis of PPM1D expression and infiltration levels of immune cells in HCC tissues based on the TIMER database.** PPM1D expression in HCC tissues positively correlates with tumor purity and infiltration levels of B cells, CD8+ T cells, CD4+ T cells, macrophages, and DCs.

Based on TIMER database, we analyzed the association of PPM1D mRNA expression with the infiltrating immune cells in 8 types of digestive tumors inclusive of HCC. These results showed the expression of PPM1D mRNA remarkably associated with tumor purity, CD8+ T cells, macrophages, CD4+ T cells, DCs and B cells of them. (Figure 3 and Supplementary Figure 2).

We also detected that higher PPM1D mRNA expression related with higher immune cells infiltration and worse prognosis in HCC (Figure 3). The PPM1D mRNA expression positively associated with the infiltration rates of the CD4+ T cells (r = 0.329, *P* = 4.06e-10), CD8+ T cells (r = 0.237, *P* = 9.15e-06), B cells (r = 0.276, *P* = 2.00e-07), macrophages (r = 0.402, *P* = 1.16e-14), and DCs (r = 0.385, *p* = 1.88e-13) in HCC tissues. These findings indicate that PPM1D is vital in immune cells infiltration in HCC, particularly in macrophages. Although these findings indicate differences in the level of tumor infiltration by the immune cell, the mRNA expression of PPM1D and prognosis in various tumors, the results generally imply that mRNA expression of PPM1D modulates the infiltration of the immune cells to cancer tissues.

### Association analysis of PPM1D mRNA expression with the marker of distinct subgroups of immune cell

The relation between PPM1D mRNA expression and the level of TIICs in HCC tissues was analysed based on the GEPIA and TIMER databases. Considering that the analysis of immune infiltration is affected by tumor purity of clinical samples, so we used purity to adjust the analysis (Table 3). The immune cells in HCC tissue including T cells (general), B cells, CD8+ T cells, TAMs, DCs, NK cells, M1 and M2 macrophages, monocytes, Tregs, Th17, Th1, Th2, exhausted T and Tfh cells were analyzed, using CHOL as the control.

**Table 3:** Correlation analysis between *PPM1D* and related genes and markers of immune cells in TIMER.

**Description**	**Gene markers**	**HCC**				**CHOL**			
		**None**		**Purity**		**None**		**Purity**	
		**Core**	***P***	**Core**	***P***	**Core**	***P***	**Core**	***P***
CD8+ T cell	CD8A	0.17	^**^	0.254	^***^	1.107	0.533	0.019	0.914
	CD8B	0.07	0.18	0.15	^**^	0.073	0.673	0.003	0.987
T cell (general)	CD3D	0.03	0.59	0.1	0.06	0.232	0.173	0.152	0.385
	CD3E	0.1	0.06	0.206	^***^	0.172	0.315	0.066	0.705
	CD2	0.08	0.12	0.182	^***^	0.151	0.377	0.046	0.791
B cell	CD19	0.18	^***^	0.222	^***^	0.307	0.068	0.243	0.160
	CD79A	0.14	^**^	0.238	^***^	0.287	0.090	0.221	0.203
Monocyte	CD86	0.27	^***^	0.392	^***^	0.127	0.460	0.023	0.897
	CSF1R	0.21	^***^	0.326	^***^	0.172	0.315	0.100	0.568
TAM	CCL2	0.19	^***^	0.295	^***^	0.281	0.096	0.246	0.155
	CD68	0.18	^***^	0.255	^***^	0.181	0.289	0.132	0.449
	IL10	0.23	^***^	0.324	^***^	0.251	0.140	0.158	0.364
Ml Macro-phage	NOS2	0.21	^***^	0.223	^***^	0.307	0.069	0.312	0.068
	IRF5	0.39	^***^	0.381	^***^	0.219	0.199	0.179	0.304
	PTGS2	0.28	^***^	0.404	^***^	0.383	0.021	0.338	0.046
M2 Macro-phage	CD163	0.28	^***^	0.393	^***^	0.392	0.018	0.343	0.044
	VSIG4	0.18	^***^	0.285	^***^	0.28	0.099	0.216	0.213
	MS4A4A	0.22	^***^	0.33	^***^	0.286	0.091	0.21	0.227
Natural killer cell	KIR2DL1	0.06	0.23	0.058	0.28	-0.202	0.237	–0.239	0.166
	KIR2DL3	0.18	^***^	0.217	^***^	-0.015	0.929	–0.04	0.818
	KIR2DL4	0.12	0.02	0.152	^**^	-0.214	0.211	–0.272	0.114
	KIR3DL1	0.13	0.01	0.157	^**^	-0.124	0.471	–0.158	0.364
	KIR3DL2	0.08	0.12	0.130	0.02	-0.105	0.542	–0.113	0.516
	KIR3DL3	0.04	0.5	0.017	0.75	-0.191	0.265	–0.225	0.193
	KIR2DS4	0.09	0.08	0.099	0.07	-0.114	0.507	–0.149	0.392
Dendritic cell	HLA-DPB1	0.16	^**^	0.253	^***^	0.088	0.608	–0.004	0.982
	HLA-DQB1	0.07	0.21	0.147	^**^	0.311	0.066	0.269	0.118
	HLA-DRA	0.22	^***^	0.321	^***^	0.071	0.681	–0.033	0.850
	HLA-DPA1	0.22	^***^	0.322	^***^	0.056	0.743	–0.048	0.783
	BDCA-1	0.19	^***^	0.263	^***^	0.136	0.428	0.052	0.767
	BDCA-4	0.51	^***^	0.541	^***^	0.515	^**^	0.484	^**^
	CD11c	0.27	^***^	0.356	^***^	0.288	0.089	0.219	0.206
Th1	TBX21	0.10	0.05	0.176	^**^	0.147	0.390	0.033	0.852
	STAT4	0.18	^***^	0.232	^***^	0.099	0.564	0.04	0.820
	STAT1	0.41	^***^	0.446	^***^	0.4	^*^	0.379	^*^
	TNF	0.12	0.02	0.187	^***^	0.037	0.83	–0.067	0.703
	INF-α	0.22	^***^	0.309	^***^	0.319	0.059	0.294	0.087
Th2	GATA3	0.16	^**^	0.280	^***^	0.046	0.79	–0.082	0.638
	STAT6	0.39	^***^	0.366	^***^	0.529	^**^	0.548	^***^
	STAT5A	0.35	^***^	0.404	^***^	0.228	0.181	0.196	0.258
	IL13	0.09	0.08	0.097	0.07	0.04	0.818	–0.011	0.95
Tfh	BCL6	0.41	^***^	0.401	^***^	0.407	^*^	0.4	^*^
	IL21	0.12	0.02	0.162	^**^	0.326	0.053	0.286	0.095
Th17	STAT3	0.49	^***^	0.528	^***^	0.547	^***^	0.557	^***^
	IL17A	0.1	0.06	0.107	0.05	0.053	0.759	0.005	0.979
Treg	FOXP3	0.3	^***^	0.335	^***^	0.264	0.12	0.189	0.277
	CCR8	0.43	^***^	0.513	^***^	0.344	^*^	0.293	0.088
	STAT5B	0.74	^***^	0.731	^***^	0.642	^***^	0.637	^***^
	TGFB1	0.21	^***^	0.297	^***^	0.307	0.069	0.265	0.124
T cell exhaustion	PD-1	0.14	^**^	0.198	^***^	0.203	0.234	0.157	0.369
	CTLA4	0.11	0.03	0.186	^***^	0.225	0.186	0.171	0.325
	LAG3	0.11	0.04	0.149	^**^	0.08	0.64	0.005	0.977
	TIM-3	0.23	^***^	0.351	^***^	0.126	0.464	0.036	0.839
	GZMB	0.04	0.43	0.075	0.17	0.039	0.823	–0.058	0.742

**Figure 4:**
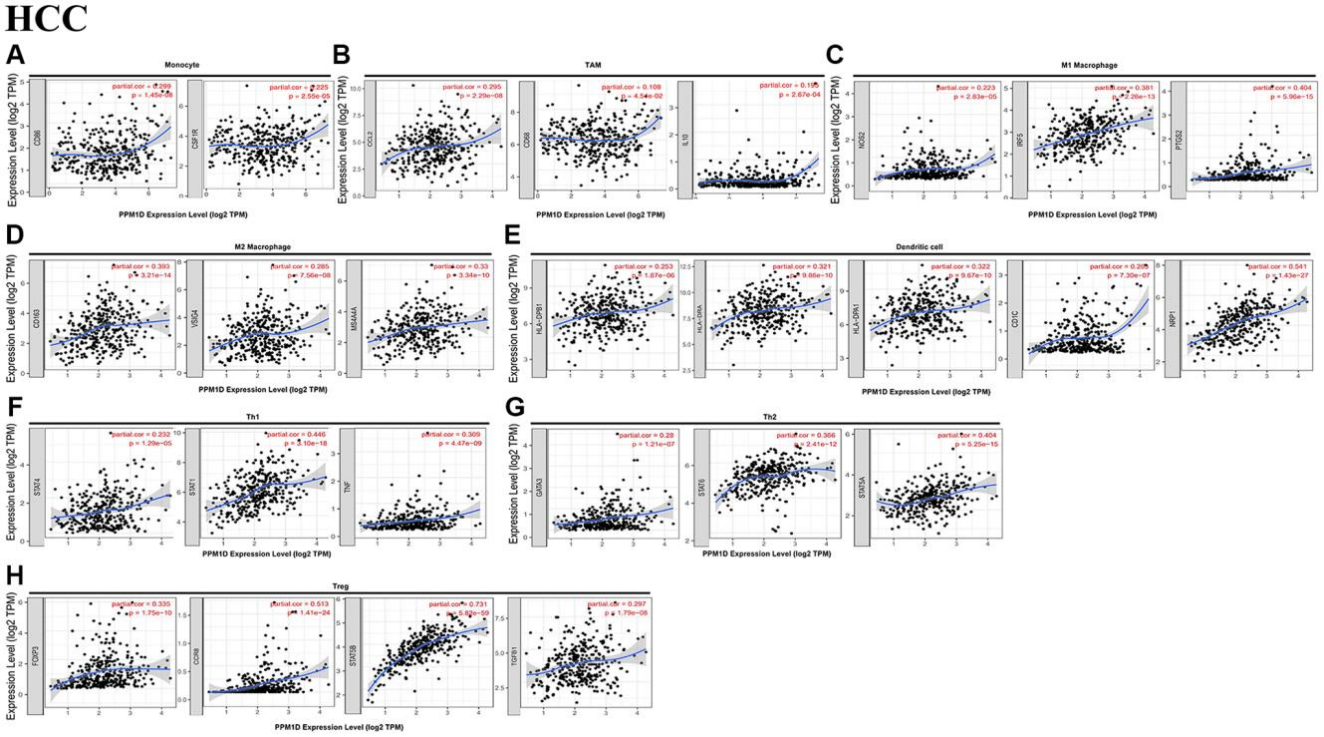
Correlation analysis of PPM1D mRNA expression and the expression of marker genes of infiltrating immune cells in HCC (**A**–**H**) using the TIMER database. (**A**–**G**) The scatter plots show correlation between PPM1D expression and the gene markers of (**A**) Monocytes (CD86 and CSF1R); (**B**) TAMs (CCL2, IL-10 and CD68); (**C**) M1 Macrophage (NOS2, IRF5 and PTGS2); (**D**)M2 Macrophage (CD163, VSIG4 and MS4A4A); (**E**) DCs (HLA-DPB1, HLA-DRA, HLA-DPA1, CD1C and NRP1); (**F**) Th1 cells (STAT4, STAT1 and TNF); (**G**) Th2 cells (GATA3, STAT6 and STAT5A) and (**H**) Tregs (FOXP3, CCR8, STAT5B and TGFB1) in HCC samples (*n* = 371). PPM1D gene was on the x-axis and the related marker genes were on the y-axis.

According to the GEPIA and TIMER databases, PPM1D mRNA expression in HCC tissue remarkably associated with the expression of marker gene due to TAMs, tumor-infiltrating Monocytes, M1 Macrophage, M2 Macrophage, DCs, Th and Treg (Figure 4 and Table 4), while the association was not remarkable in CHOL (Supplementary Figure 3 and Table 4).

**Table 4:** Correlation analysis between PPM1D and related genes and markers in GEPIA.

**Description**	**Gene markers**	**HCC**				**CHOL**			
		**Tumor**		**Normal**		**Tumor**		**Normal**	
		***R***	***P***	***R***	***P***	***R***	***P***	***R***	***P***
Monocyte	CD86	0.28	^***^	0.55	^***^	0.13	0.44	0.60	0.097
	CD115	0.25	^***^	0.52	^***^	0.20	0.25	0.23	0.55
TAM	CCL2	0.18	^***^	0.23	0.11	0.25	0.14	0.55	0.13
	CD68	0.21	^***^	0.57	^***^	0.21	0.21	0.43	0.25
	IL10	0.25	^***^	0.39	^**^	0.29	0.08	0.38	0.32
Ml Macrophage	iNOS	0.25	^***^	0.14	0.33	0.37	^*^	–0.10	0.80
	IRF5	0.38	^***^	0.26	0.07	0.21	0.22	0.25	0.52
M2 Macrophage	VSIG4	0.17	^**^	0.43	^**^	0.30	0.08	0.05	0.91
	MS4A4A	0.22	^***^	0.50	^***^	0.30	0.08	0.13	0.74
Dendritic cell	HLA-DPB1	0.18	^***^	0.45	^***^	0.10	0.57	0.13	0.74
	HLA-DQB1	–0.05	0.38	0.20	0.17	0.24	0.16	0.20	0.61
	HLA-DRA	0.22	^***^	0.47	^***^	0.042	^*^	0.18	0.64
	HLA-DPA1	0.22	^***^	0.46	^***^	0.026	0.88	0.12	0.78
	BDCA-1(CD1C)	0.18	^***^	0.31	^*^	0.073	0.67	0.05	0.91
	CD11c(ITGAX)	0.30	^***^	0.40	^**^	0.30	0.07	0.62	0.09

“R” is a correlation coefficient, the higher the value, the stronger the correlation.

The mRNA expression of PPM1D revealed that the association with the expression of the markers of particular immune cell including the monocyte marker, such as CD86 (r = 0.392; *P* = 3.77e-14), IRF5 (r = 0.381; *P* = 2.26e-13), COX2 (r = 0.404; *P* = 5.96e-15), CD163 (r = 0.393; P = 3.21e-14), BDCA-4 (r = 0.541; *P* = 1.43e-27), STAT1 (r = 0.446; *P* = 3.10e-18), STAT6 (r = 0.366; *P* = 2.41e-12), STAT5A (r = 0.404; *P* = 5.25e-15), CCR8 (r = 0.513; P = 1.41e-24), and STAT5B (r = 0.731; *P* = 5.82e-59) (Table 1). These findings imply that PPM1D mRNA expression correlated with infiltration of the immune cell in HCC.

### Immunohistochemical examination about the expression of Wip1 in HCC samples

**Figure 5:**
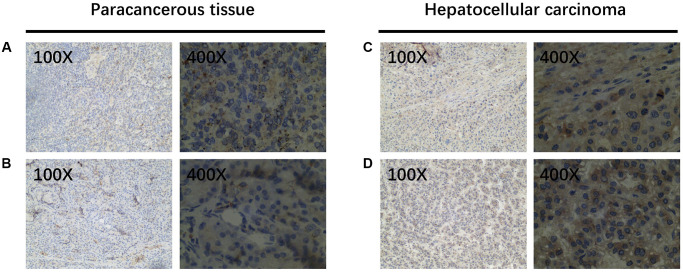
**WIP1 expression in paracancerous and hepatocellular carcinoma tissues.** (**A**, **B**) Wip1 is low expressed in paracancerous tissues; (**C**, **D**) Wip1 is highly expressed in tumor tissues.

We collected 40 patient specimens for immunohistochemical examination and completed the follow-up work. All the patients were divided into two groups with good prognosis and poor prognosis by the median survival time. Then the expression levels of Wip1 were analyzed in the two groups. Wip1 was highly expressed in hepatocellular carcinoma compared with precancerous tissue (Figure 5). There were 6 patients with high wip1 expression in good prognosis group, and 15 patients with high wip1 expression in poor prognosis group. In summary, higher expressions of Wip1 were intently correlated to worse prognosis, as shown in Table 5.

**Table 5:** The relationship between Wip1 expression and prognosis in HCC.

**Prognosis**	**Wip1 expression**		***χ*^2^**	***P* value**
	**Low**	**High**		
Good prognosis	14	6	8.120	0.004
Poor prognosis	5	15

## DISCUSSION

This study illustrated that mRNA expression of PPM1D were correlated with several human cancer prognosis. High PPM1D mRNA levels correlated with poor prognosis in HCC. Furthermore, PPM1D mRNA levels associated with the level of TIICs, with respect to the status of the markers for the various immune cell types in HCC. These findings suggested that PPM1D is an underlying prognostic marker for HCC and other tumors.

The Sangerbox, TIMER, GEPIA, and Kaplan-Meier Plotter databases were used to study the status of PPM1D mRNA in tumor tissue. The analysis demonstrated that PPM1D expression was significantly upregulated in most cancers. However, the expression of PPM1D differed in various cancers. This reflects differences in different methods of data collection as well as hidden causative mechanism. Furthermore, our results of PPM1D expression was consistent in HCC tissues with other databases. Moreover, we also found PPM1D protein was upregulated in HCC tissue than the paracancerous tissue. The GEPIA database gene expression analysis revealed that higher PPM1D expression related with worse prognosis in cancers including HCC, BLCA, CESC, MESO and UVM. Besides, Kaplan-Meier Plotter analysis disclosed that PPM1D higher expression related with worse prognosis in HCC. Through immunohistochemistry, we also verified the high expression of PPM1D protein. The high PPM1D expression related with poor prognosis of the patients in early stage (stage 1 and AJCC_1), and long PFS and OS in HCC patients with low PPM1D expression (Table 1). The findings imply that PPM1D is one of underlying prognostic markers for HCC, especially for patients in early stage.

Dissimilar to traditional tumor treatments such as chemotherapy and radiotherapy, immunotherapy is an innovative treatment method that dynamically regulates the immune system to attack tumor cells in diverse targets and directions [20]. In various basic experiments and clinical researches, it has been found that immunotherapy does have an incomparable advantage over traditional cancer treatments, and can prolong OS and PFS [21]. Current research shows that immunotherapy also plays a significant role in HCC [22, 23]. In recent years, immune checkpoint inhibitors (anti-CTLA-4, anti-PD-1 and anti-PD-L1 antibodies) have shown therapeutic potentiality on advanced HCC [24, 25]. Therefore, an in-depth understanding of tumor immune infiltration can help provide prognostic predictors for immunotherapy, and provide more precise and individualized treatment for HCC.

A previous study showed that a PPM1D-deficient mouse has defective immune response [26]. There was cumulative evidence suggested that a PPM1D-deficient mouse has immune response deficiency [27], neutrophil migration and development via p38-MAPK-STAT1 pathway [28]; macrophage migration and phagocytosis [29]; dendritic spine memory and morphology via p53MAPK signaling. PPM1D mutations assembled in H3F3A-mutated malignant brainstem gliomas, and high intratumoral CD8+ T cell density was less common in the H3F3A-mutated tumors. Moreover, patients with H3F3A-mutated tumors experienced worse prognoses in comparison to other patients. It is speculated that PPM1D may be related to poor prognosis resulting from low intracranial CD8+ T cells [30].

Our study demonstrates that higher PPM1D expression correlated with worse prognosis in HCC and infiltration of different types of immune cell inclusive of CD4+, CD8+ T cells, macrophages, B cells and DCs. As an important component of the tumor microenvironment, TAM play a vital role in immune regulation. The increase of TAM and PD-L1 in liver cancer shows the characteristics of immune escape [31]. We established the relationship between expression of PPM1D mRNA and TAM markers, IL-10 and CD68, and iNOS, M1 macrophage marker, IRF5 and COX2. This implies that PPM1D activates the infiltration and TAM’s activity. PPM1D expression was also associated with the expression of the markers various subsets of Th cells, including Tregs (FOXP3, STAT5B and TGF-β), Tfh (BCL6), and Th1 (STAT-1, STAT-4 and TNF-α), Th2 (GATA3, STAT6, STAT35A). This suggests the vital contribution of the PPM1D in adjusting tumor-infiltration of the Th cells. Besides, the expression of the deficient T cells marker, TIM-3 and PD-1, which were critical inhibitory immune checkpoint proteins definitely associated with the PPM1D expression. Interestingly, PPM1D is highly expressed in HCC and CHOL from Sangerbox and TIMER database., and our previous researches have illustrated that PPM1D is associated with poor prognosis of cholangiocarcinoma [11]. However, the analysis found that PPM1D is not correlated to the immune cell infiltration of CHOL, suggesting that the association of PPM1D on immune cell infiltration has a special role in HCC, which is worthy of further study.

Currently, researchers are concerned about the contribution of PPM1D in HCC. It is reported that mRNA of PPM1D was remarkably higher in HCC than corresponding normal tissue from 86 HCC. The high PPM1D was related to TNM stage, alpha- fetoprotein (AFP) level, tumor size, recurrence incidence and the family history for HCC. Nevertheless, PPM1D expression and age, gender, alcohol intake, lymph node metastasis, hepatitis B virus infection or portal vein invasion did not show any correlation [32]. In this study, we realized that the upregulation of PPM1D correlated with poor prognosis in specific patients who had some specific clinical characteristics inclusive of alcohol consumers, Asians, males, those in early stages (stage 1 and AJCC_1), and the suffering from hepatitis viral infections. The findings in this study consistent with our previous study [11, 14]. However, the credibility of this study could be higher than that of a single-center study since it emanates from multiple databases. In general, our findings imply that a high expression of PPM1D could be related with poor prognosis of HCC.

Final note: Our research has some limitations. The first point is that many of our data sources are previously reported data, not all of our own clinical data. The second point is that some tumors in the database have too few samples.

## CONCLUSIONS

Our findings suggest that PPM1D might be an underlying prognostic marker for HCC, that could be taken to determine the rate of immune cells infiltration in cancer tissue. The comparatively high levels of PPM1D in HCC and other cancers imply a high risk for tumor relapse after treatment. As such, close medical monitoring is vital for these patients.

## Supplementary Materials

Supplementary Figures
