# Supplementary material for: PPM1D is a potential prognostic biomarker and correlates with immune cell infiltration in hepatocellular carcinoma
Source: Aging (Albany NY). 2021 Sep 1;13(17):21294–308. doi: 10.18632/aging.203459 (PMC8457582; doi:10.18632/aging.203459)
Supplement: Supplementary Figures [file aging-13-203459-s001.pdf]

SUPPLEMENTARY FIGURES

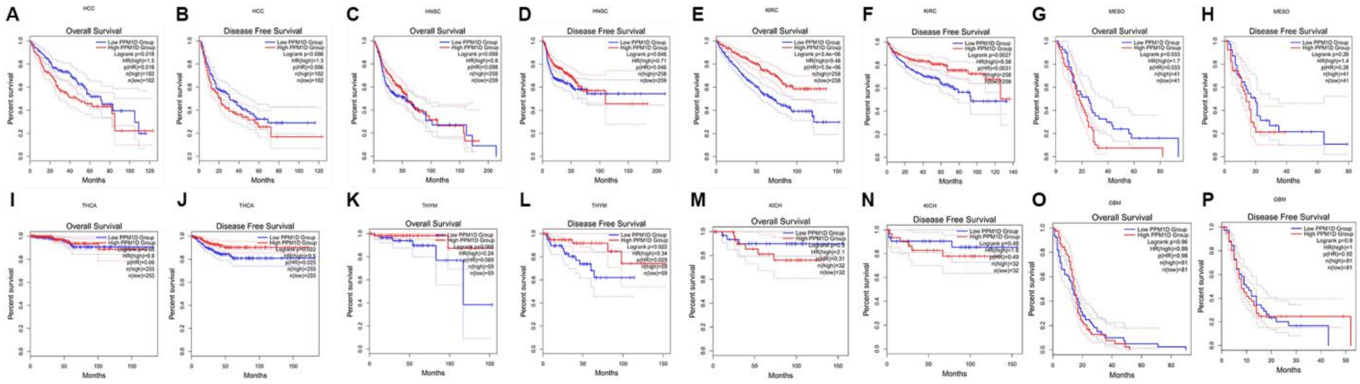

Supplementary Figure 1. Correlation of PPM1D mRNA expression with prognostic values in diverse types of cancer in the GEPIA databases. Overall survival and disease free curves comparing the high and low expression of PPM1D in Hepatocellular carcinoma (HCC) (**A**, **B**), Head and Neck squamous cell carcinoma (HNSC) (**C**, **D**), Kidney renal clear cell carcinoma (KIRC) (**E**, **F**), Mesothelioma (MESO) (**G**, **H**), Thyroid carcinoma (THCA) (**I**, **J**), Thymoma (THYM) (**K**, **L**), Kidney Chromophobe (KICH) (**M**, **N**), Glioblastoma multiforme (GBM) (**O**, **P**).

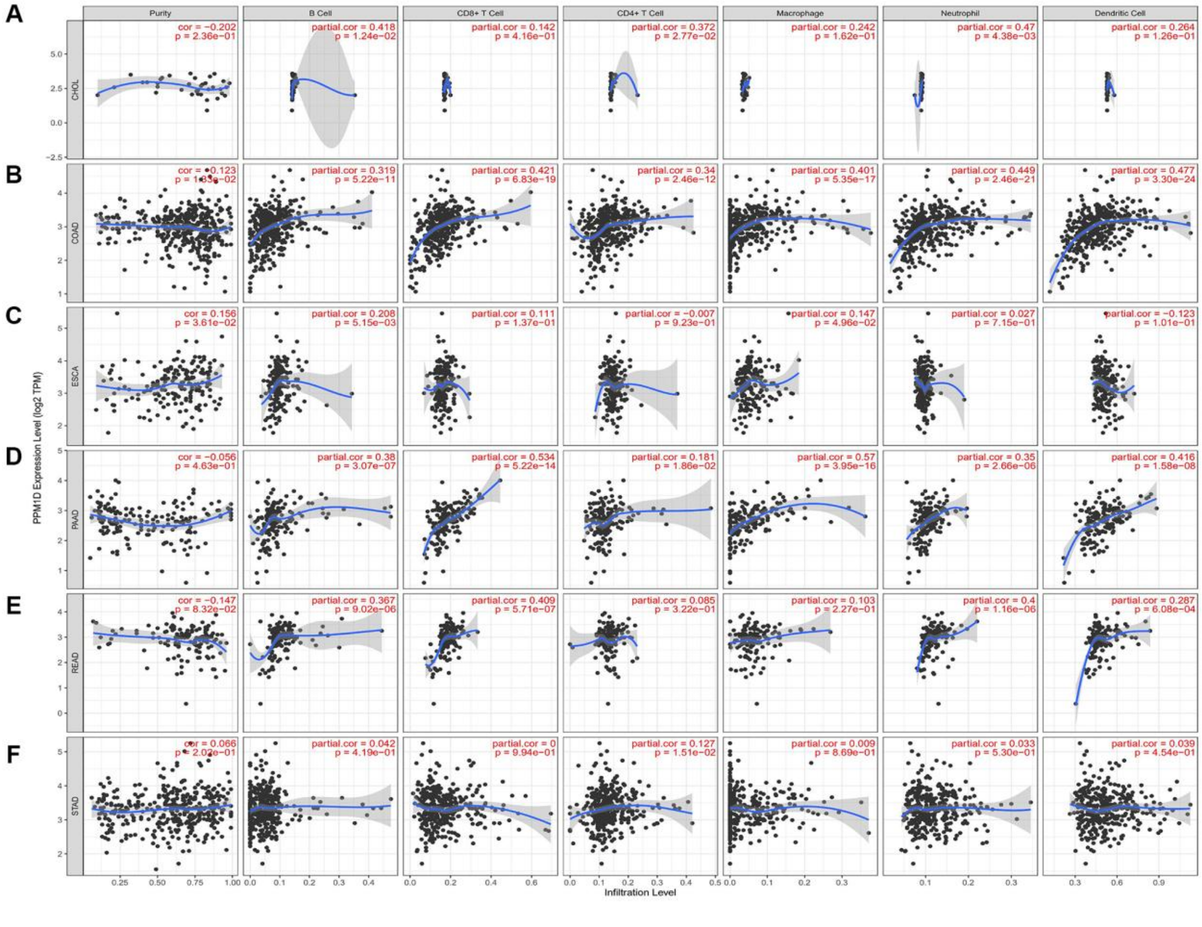

Supplementary Figure 2. Correlation analysis of PPM1D expression and infiltration levels of immune cells in digestive system tumors tissues based on the TIMER database. PPM1D expression in HCC tissues positively correlates with tumor purity and infiltration levels of B cells, CD8+ T cells, CD4+ T cells, macrophages, and DCs. (**A**) Cholangiocarcinoma (CHOL), (**B**) Colon adenocarcinoma (COAD), (**C**) Esophageal carcinoma (ESCA), (**D**) Pancreatic adenocarcinoma (PAAD), (**E**) Rectum adenocarcinoma (READ), (**F**) stomach adenocarcinoma (STAD).

CHOL

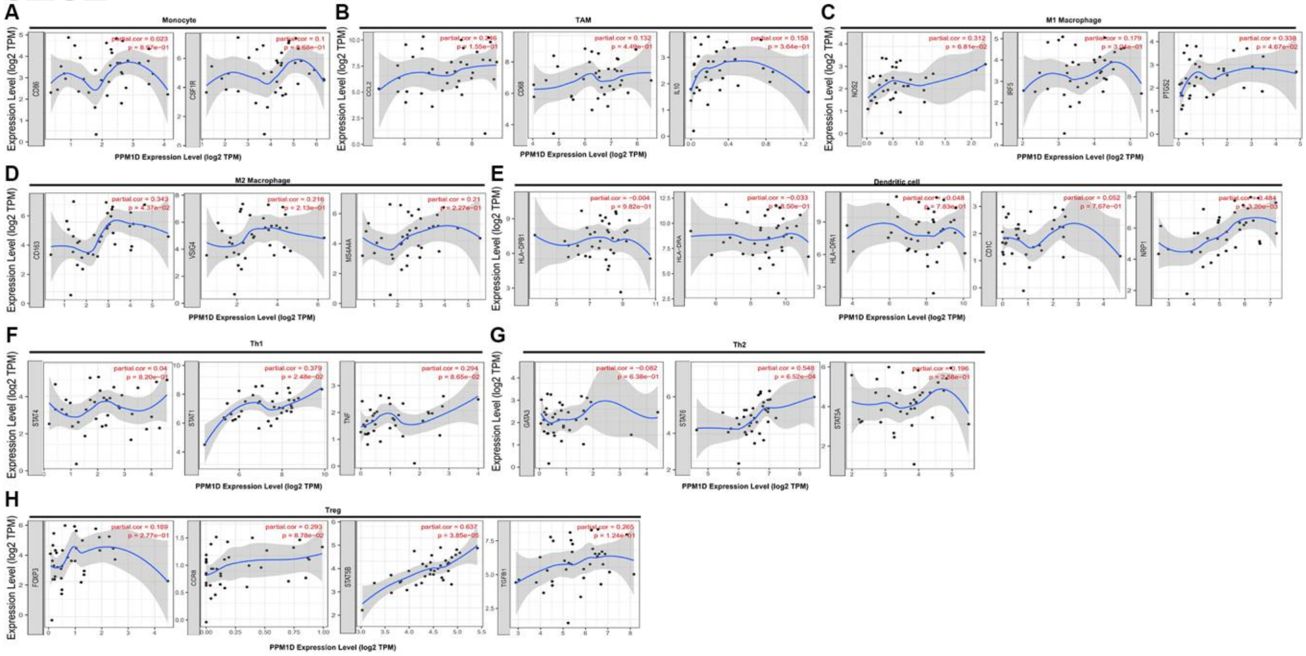

Supplementary Figure 3. Correlation analysis of PPM1D mRNA expression and the expression of marker genes of infiltrating immune cells in CHOL (**A**–**H**) using the TIMER database. (**A**–**G**) The scatter plots show correlation between PPM1D expression and the gene markers of (**A**) Monocytes (CD86 and CSF1R); (**B**) TAMs (CCL2, IL-10 and CD68); (**C**) M1 Macrophage (NOS2, IRF5 and PTGS2); (**D**) M2 Macrophage (CD163, VSIG4 and MS4A4A); (**E**) DCs (HLA-DPB1, HLA-DRA, HLA-DPA1, CD1C and NRP1); (**F**) Th1 cells (STAT4, STAT1 and TNF); (**G**) Th2 cells (GATA3, STAT6 and STAT5A) and (**H**) Tregs (FOXP3, CCR8, STAT5B and TGFB1) in HCC samples (*n* = 36). PPM1D gene was on the x-axis and the related marker genes were on the y-axis.
